# PNS for management of intercostal neuralgia: A case report^☆^

**DOI:** 10.1016/j.inpm.2025.100573

**Published:** 2025-03-18

**Authors:** Christopher Morris, Kevin Vorenkamp, John Ward

**Affiliations:** Duke University Hospital, USA

## Abstract

Intercostal neuralgia is a rare but potentially debilitating condition that manifests as neuropathic pain in any rib space. This pain can typically be treated with typical mainstays of neuropathic pain treatment, such as over-the-counter analgesics, gabapentinoids, serotonin-norepinephrine reuptake inhibitors, tricyclic antidepressants, and opioids. However, as detailed in this case, patients can have refractor pain despite the use of these mainstays of treatment. In these patients, peripheral nerve stimulator (PNS) placement can be a possible treatment modality. In our case detailing a 75 year old male with refractory intercostal neuralgia, we have shown that PNS placement for this indication can provide analgesia in this debilitating condition.

## 1 Introduction

Intercostal neuralgia is a rare and potentially debilitating condition characterized by neuropathic pain in any rib space. The mainstays of treatment are that of typical neuropathic pain, including over-the-counter analgesics, gabapentinoids, serotonin-norepinephrine reuptake inhibitors, tricyclic antidepressants, and opioids. When these therapies are not providing adequate analgesia to the patient, peripheral nerve stimulator (PNS) implantation can be considered, although limited data exists to provide detailed patient outcomes. PNS have had reported success in the treatment of various refractory cases of chronic pain including but not limited to chronic headaches, pelvic pain, low back pain, postamputation pain, and lower extremity pain [4]. Detailed in this case report is a 75 year old man with refractory intercostal neuralgia and exclusively anterior chest wall pain, who has had improvement of his symptoms following PNS placement. Due to the anterior chest wall location of his pain, an anterior lead placement was performed rather than the more traditional posterior-lateral location. The patient was consented prior to the writing and publishing of this case report by IRB guidelines.

## 2 Case

A 75 year old male presented to the ambulatory pain clinic with progressive anterior chest pain in the third and fourth intercostal space. He reported the pain had been present for at least the last 10 years, but had progressively worsened over the last year. He described a sharp and burning pain, which is worsened with cold air and movement, and improves with heat. He has had trials with multiple medications, which had not provided adequate analgesia for him to sustain normal daily function. He was diagnosed with intercostal neuralgia based on his symptoms although no clear etiology was identified. In light of the patient's prior medication trials without significant improvement, PNS implant was discussed with the patient as a possible treatment modality, which the patient voiced an interest to. Prior to PNS being implanted, he was trialed with intercostal nerve block via an anterior approach, which initially provided substantial relief, but quickly returned to patient's baseline pain. Given his significant response to the block, a decision was made to move forward with PNS placement.

### 2.1 Brief op note

**Fig. 1:**
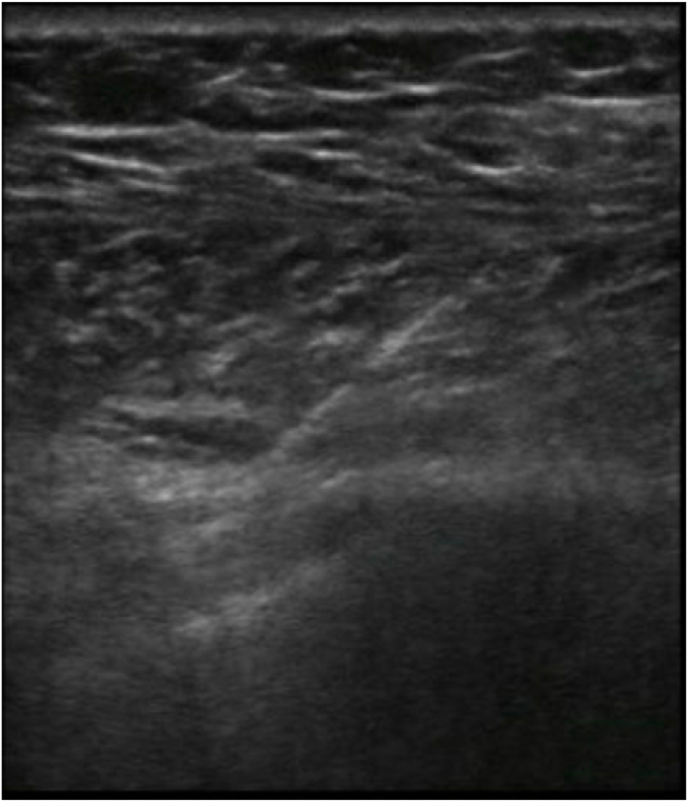
Ultrasound of lead placement.

**Fig. 2:**
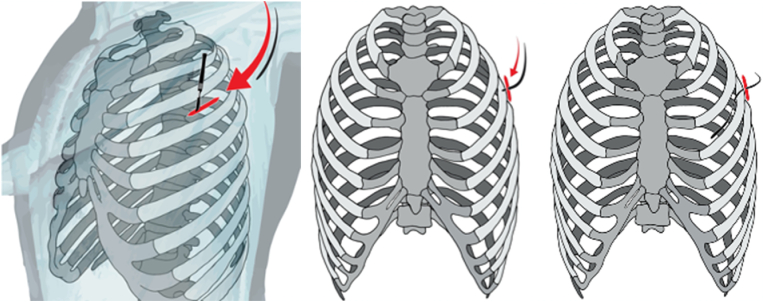
Image depiction of PNS placement.

**Fig. 3:**
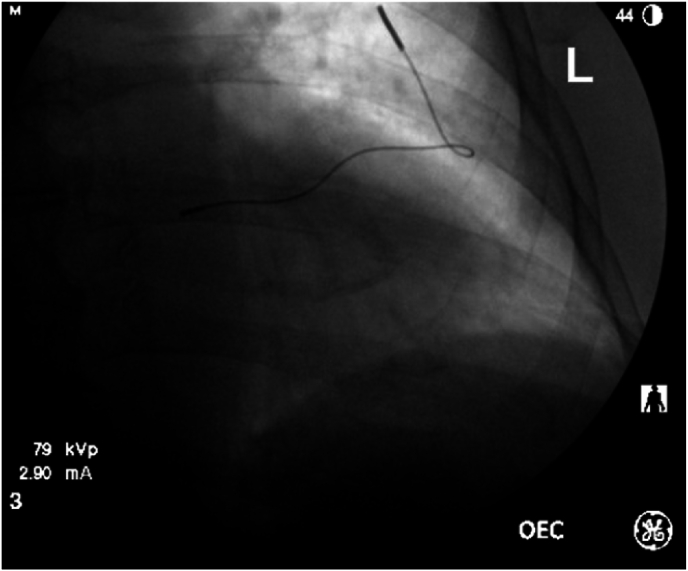
Final fluorscopic image of PNS placement.

Patient was placed in supine position, and with ultrasound guidance, relevant anatomy was identified. The patient was anesthetized with local lidocaine, and a small incision was made for lead placement and tunneling between the third and fourth intercostal spaces. Through this incision, and with ultrasound guidance, a guidewire was advanced until the fascia deep to the pectoralis major was entered (Fig. 1). Subsequent testing resulted in paresthesia in the area which the patient reported his pain. The area was then dilated, and the Bioness StimRouter lead (Bioness: 25103 Rye Canyon Loop, Valencia, CA 91355) was deployed after confirming the amplitude twitch of 0.1–0.2 milliamp. The IPG was then tunneled in a slightly more cephalad position at a depth of <1 cm from the skin, and after some manipulation, was secured with a single suture and skin glue (Fig. 2). Final positioning of the PNS lead was confirmed via fluoroscopy (Fig. 3).

The patient reported 50 % relief the day after placement, and was seen in clinic 14 days following placement of PNS, and reports that the pain had decreased to 4/10 from the 8/10 he initially presented to the clinic with. On follow-up 2 years after placement, the patient continues to report improved pain, now to 3/10 in severity.

## 3 Discussion

In this patient, since his pain was isolated to the anterior chest wall, an anterior approach was taken to target the anterior cutaneous branch of the intercostal nerve. More commonly with intercostal neuralgia, a posterior-lateral target is utilized to target the nerve before it splits into anterior and posterior branches. The intercostal PNS implant procedure is overall safe, and does have positive outcomes, as is the case with this patient, and other listed case reports [1,2]. The American Society of Pain and Neuroscience does additionally list intercostal neuralgia as an indication for PNS placement [3]. The success of the implant relies upon appropriate diagnosis and treatment targeting the nerve most responsible for patient's symptoms.

## 4 Conclusion

As illustrated in the above case, peripheral nerve stimulation can help to provide further relief to patients with intercostal neuralgia with refractory pain after trials with typical neuropathic pain medications. Although data is limited on the overall efficacy in PNS placement for intercostal neuralgia, it has been proven to be safe and effective, and warrants further investigation for patients with refractory pain.

## Declaration of competing interest

The authors declare that they have no known competing financial interests or personal relationships that could have appeared to influence the work reported in this paper.
